# American Ophthalmological Society

**Published:** 1888-10

**Authors:** 


AMERICAN OPHTHALMOLOGICAL SOCIETY.

SEPTEMBER 19.

Professor HENRY W. WILLIAMS, of Boston, President pro tempore.

Professor DAVID WEBSTER, of New York, read a paper upon "Some Tenotomies for the Correction of Heterophoria, with Results."

He reported forty cases; twenty-five had been operated on but once, in sixteen a second operation was done, and three had previously been operated on by others, making a total of sixty tenotomies. The operations had been done since the beginning of July, 1886. A slight over-correction was usually aimed at and attained. In three cases a slight reduction of the effect was necessary. All operations were done under cocaine, and the eyes tested from time to time to determine when sufficient effect had been produced. In three hysterical males remarkably good results were obtained. Most of the operations were done for headache and asthenopia. The writer had reached the conclusion that no person should have a tenotomy done for heterophoria, without inconvenience probably due to it; very slight degrees may cause trouble and should be attended to. All other methods should be tried before tenotomy.

Dr. SAMUEL THEOBALD, of Baltimore, read a paper entitled "Is Astigmatism a Factor in the Causation of Glaucoma?" The author's experience led him to believe that when the meridian of least refraction was vertical, or approached the vertical, that more effort was made to correct the difficulty, greater hyperæmia of the ciliary muscle resulted, and the condition known as glaucoma precipitated.

Dr. PETER A. CALLAN, of New York, read a paper on "The Treatment of Ulcers of the Cornea."

The ulcers occurring in young persons and children are really phlyctenulæ of the cornea, or neglect of lid friction causing absorption which gives rise to an ulcer. The treatment is yellow oxide of mercury salve (two to ten per centum) placed between the lids once daily; atropia and cocaine if necessary; tonics, open air exercise, regulation of diet, airy sleeping quarters, smoked glasses, avoidance of dark room or bandages.

He gives preference to the circumscribed application of a 2 per centum solution of nitrate of silver to the ulcer alone.

Dr. EDWARD JACKSON, of Philadelphia, read a paper on "Meridional Astigmatism." By this was meant the difference in refractive power of the same meridian as examined at the centre of the pupil or at the margin. He found considerable difference in this respect in different individuals, and thought a good deal of the difference which was found in the same individuals, according as they were or were not under the influence of atropia, due to this factor. There was some objection made to the term used by the author, as it is impossible to exclude the feature of dispersion.

The committee, composed of Doctors Edward Jackson, Henry D. Noyes, and Swan M. Burnett, appointed to consider the proposition to designate prisms according to their refractive power recommended the indorsement of the following propositions:

*First.* Prisms ought to be designated by the number of degrees of minimum deviation they produce.

*Second.* Where intervals of less than one degree are desired half degrees and quarter degrees should be used.

*Third.* To indicate that degrees of deviation are meant, the letter "d" shall be added. Thus, "prism 2°d" will indicate a prism that produces a minimum deviation of two degrees.

Dr. J. O. TANSLEY, of New York, exhibited the ordinary long box with glass sides and lenses, which, when filled with smoke, shows, by the aid of the lantern, the general principles of refraction and reflection.

Dr. W. F. MITTENDORF, of New York, read a paper on "Acute Cocaine Conjunctivitis." The notes of three cases were recorded. The affection was characterized by a swollen, shiny appearance of the conjunctiva associated with profuse acrid discharge. It was shown by the use of different salts of the same alkaloid and the special care taken with the preparation of the solution that the irritation was due to the cocaine itself. Several members had recognized similar conditions in certain cases after the use of cocaine solutions. Dr. Mittendorf suggested that it may be due to the influence of the cocaine in paralyzing the terminal fibres of the sympathetic.

Professor S. M. BURNETT, of Washington, exhibited some apparatus for diagnosis of refraction. This consisted of a disc of lenses to be used in applying the shadow test, arranged to be fixed to the wall and adjustable to any height, and readily used for either eye, or swung out of the way when not in use.

The object of the device is to facilitate the process of skiascopy. Attached to the centre of the disc is a tape-measure to determine the distance at which the observation is made.

Dr. CARL KOLLER, of New York, read a paper on "Blepharospasm." He referred especially to that form of blepharospasm which was a reflex irritation of the terminal nerve fibres in the cornea and conjunctiva. Such cases often revealed a fissure situated in a fold of skin which extends outward from the external canthus. The chief object in view in the treatment of such cases should be the relief of the external fissure.

Dr. R. H. DERBY reported a case of "Recurrent Monocular Optic Neuritis," with recovery. The remedies used were iodide of potassium and mercurial inunctions.

Dr. O. D. POMEROY reported his experience with the bident of the late C. R. Agnew, using by preference the modification suggested by Dr. Andrews. He spoke very favorably of the instrument and gave a word of caution against pushing the lens too far forward. He extracts the lens with a sharp hook or spoon.

